# A Review on Natural Fiber Bio-Composites, Surface Modifications and Applications

**DOI:** 10.3390/molecules26020404

**Published:** 2021-01-14

**Authors:** Mohammed Zwawi

**Affiliations:** Department of Mechanical Engineering, Faculty of Engineering, King Abdulaziz University, Rabigh 21911, Saudi Arabia; mzwawi@kau.edu.sa

**Keywords:** natural fibers, surface modifications, renewable, bio-degradable

## Abstract

Increased environmental concerns and global warming have diverted focus from eco-friendly bio-composites. Naturals fibers are abundant and have low harvesting costs with adequate mechanical properties. Hazards of synthetic fibers, recycling issues, and toxic byproducts are the main driving factors in the research and development of bio-composites. Bio-composites are degradable, renewable, non-abrasive, and non-toxic, with comparable properties to those of synthetic fiber composites and used in many applications in various fields. A detailed analysis is carried out in this review paper to discuss developments in bio-composites. The review covers structure, morphology, and modifications of fiber, mechanical properties, degradable matrix materials, applications, and limitations of bio-composites. Some of the key sectors employing bio-composites are the construction, automobile, and packaging industries. Furthermore, bio-composites are used in the field of medicine and cosmetics.

## 1. Introduction

Increased focus is being placed on the need to reduce global warming, environmental damage, and pollution. The scientific community has been paying significant attention to developing environmentally friendly and bio-degradable materials that can replace the non-renewable materials that pose a threat to the environment [1,2]. Bio-composite materials have become the center of attention due to their environmentally friendly and biodegradable nature [3,4]. A number of hazards and shortcomings are associated with synthetic composites. They have larger carbon footprints and need a large amount of energy for fabrication [5]. A variety of inorganic fibers, including nylon, Kevlar, polypropylene, and glass, are used in synthetic composites [6]. Fossil fuel depletion also endangers the sustainability of these synthetic materials in the long term [7].

The dangers of climate change have made us focus more on reducing global warming, which made many developed countries pledge to limit the increase in average worldwide temperature below 2 °C [8]. Unlike synthetic materials, bio-composites can be degradable without emissions of poisonous gases or substances [9]. Microbes degrade biomaterials into an organic substance through compositing along with the release of minerals, water, and CO_2_ [10,11]. Industries are encouraged to use bio-friendly materials to have a better impact on the environment [12]. These bio-composite materials are promising candidates to overcome contemporary environmental issues, [1] reduce energy demand, [13] and to reduce carbon footprints [14,15]. On average 17% less energy is required to produce natural composites than synthetic counterparts [7].

The global bio-composite market’s projected growth rate is 9.59%, to reach a USD 41 billion net worth by 2025 [16]. The automobile and construction industries are two major sectors for bio-composites. Bio-composites are eco-friendly, degradable, renewable, non-abrasive, non-toxic, and have low densities [17]. These materials are used in cars to reduce the overall weight and to enhance fuel efficiency. Bio-composites are utilized to manufacture door panels, armrests, seatbacks, and trays [2]. They are also used externally for trim parts and brake shoes. Bio-composite parts are better at sound absorbance and shatter resistance [18].

Fibers used in bio-composites are produced from agricultural products and byproducts, which are subsequently intermixed with different polymer-based matrices [19]. Biodegradable and renewable polymer matrices are mixed with natural fibers known as lignocellulosic fibers [20]. Natural fibers are mostly used as reinforcements but also can be used as matrix material [21,22]. Bio-composites fall under the category of polymer matrix composites. Polymer matrix composites are made up of natural (PLA, PHA, PCL) or synthetic matrix materials (thermoplastic, thermosetting plastic), with one or more reinforcements such as carbon fibers, glass fibers or natural fibers in the case of bio-composites [23,24]. Cellulose fibers are organic and are produced from biomass [25] and associated derivatives of agricultural products [26]. Cellulose is currently considered one of the most studied and used polymers, followed by lignin [27]. Approximately 40–60% of plant matter consists of cellulose, in addition to hemicellulose, lignin, and pectin [28]. The basic cellulose unit is anhydro-d-glucose, which contains three hydroxyls responsible for hydrophilic nature [29]. Cellulose offers superior mechanical properties, while lignin reduces water sorption and enhances thermal stability [30]. Lignin serves to bind plant parts together, thereby acting as a cementing material. It also influences the structure and properties of plants [20]. The lumen is a hollow central cavity in a fiber cell, responsible for reducing the density, increasing thermal insulation, and noise-resistance properties [31]. Microfibril is a primary structural unit in the cell wall of a plant. The angle at which the microfibril fiber connects with the cell wall directly influences the mechanical properties and acts as a reinforcing element arising from the linear linkage of crystallites [32]. Certain types of lignocellulosic fibers exhibit mechanical properties and overall strength comparable to that of synthetic fibers such as fiberglass [33].

Thermoplastic polymer matrices, such as polypropylene and polyethylene, are hydrophobic and offer low compatibility with natural fibers. Surface treatments decrease the fibers’ surface energy to optimize the strength and properties of the composite [34]. Bio-composite performance is ultimately dependent on the fiber/matrix interphase. Adhesion between the matrix and fiber determines the final properties of the composite [35]. The mechanical properties of a composite depend on the amount and type of filler being used, how fiber adheres to the material, and the final fiber orientation in the matrix [36]. The properties of these lignocellulosic fibers are also dependent on the origin of the plant species, fiber, location of the plant, environment around the plant, and methods to extract the fibers [20].

Polybutylene succinate (PBS), polylactic acid (PLA) [9,37], poly hydroxyalkanotes (PHA) [38], and poly(*ε*-caprolactone) (PCL) [39] are commonly used biodegradable matrices in bio-composites. Synthetic matrix materials are not biodegradable. Some synthetic matrix materials are polyethylene, polypropylene, polycarbonate, polyvinylchloride, nylon, acrylics, and carbon steel Kevlar, epoxy resins, etc. [40]. Out of these, due to its eco-friendly and degradable nature, PLA has attracted significant attention. PLA is synthesized via direct starch fermentation. The use of a ring-opening approach to polymerize cyclic lactide dimers is preferred for PLA with a higher molecular weight. PLA is crystalline, transparent, and brittle in nature [9]. PHA is generally produced using a microbial process in carbon substrate, and it degrades easily at room temperature. However, it has mostly limited use due to the high cost [38]. PBS belongs to aliphatic polyesters and is produced by two-step polycondensation. PBS is semi-crystalline with an aliphatic structure and is biodegradable due to the presence of odd ester bonds. However, like PLA, it has a higher production cost [41,42]. PCL is developed from crude oil through the ring opening polymerization of caprolactone monomers [39]. The action of microorganisms degrades it with water, CO_2_, minerals, and methane. PLA exhibits inferior properties in comparison with PBS and PCL, with higher production costs [43].

Green bio-composites have pros and cons. Limitations of bio-composites include poor fire resistance [2], restricted processing temperature, low thermal resistance [32], high hydrophilicity, low mechanical and thermo-physio properties [40], and poor fiber–matrix adhesion [44,45]. Due to their hydrophilic nature, these composites tend to absorb water from the immediate environment [32], causing the composite to swell. Stem fiber, leaf fiber, and seed fiber are the three main fibers [20]. The most common natural fibers are hemp, doum, coir, jute, almond shells, rice husk, oat husk, wheat straw, switchgrass, corona, kenaf, coconut, bamboo, bagasse, banana, sisal, sugarcane, oil palm empty fruit bunch [20,46,47,48,49,50,51,52,53,54,55,56,57,58,59,60,61].

## 2. Lignocellulosic Fibers

**Figure 1:**
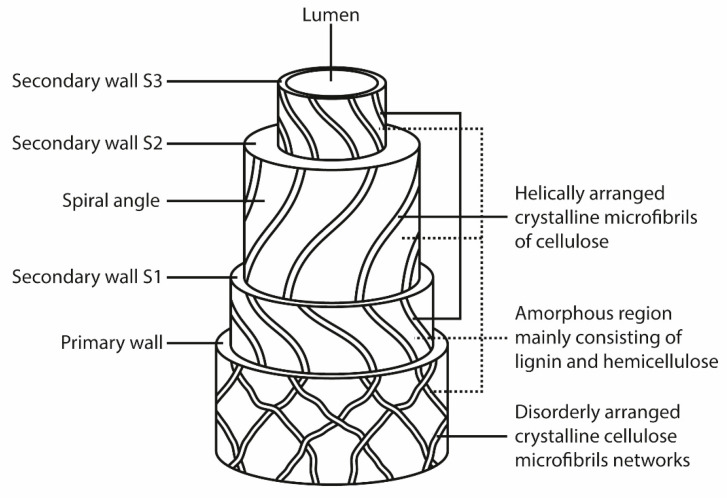
Shows structural constituents of a natural plant fiber. Reprinted with the permission from the publisher [64].

Lignocellulosic fibers consist of cellulose, hemicellulose, lignin, pectin, waxes, extractive, and trace elements [62,63]. Figure 1 shows the different structural constituents of a plant fiber.

### 2.1. Cellulose

Cellulose is the most abundant form of living terrestrial organism. Purer forms of cellulose include cotton and hemp fibers, while in wood, stalks and leaves, cellulose is found in combination with lignin and hemicellulose. Apart from plants, some bacteria and fungi are found to have cellulose as well. In plants, cellulose is located in a secondary wall that consists of linear homopolysaccharide composed of anhydro-d-glucose (C_6_H_11_O_5_). Linkages of 1,4-*b*-d-glycosidic join them with a degree of polymerization near 10,000, and each repeating unit contains three hydroxyl groups. Cellobiose is a repeating unit and a dimer of glucose [65]. Hydrogen bonding between hydroxyl groups (with water elimination [66]) is responsible for the 3D crystallinity of the structure and the hydrophilic nature [29,64,67]. The length of the 1,4-*b*-d-glycosidic linkages is dependent on the cellulose source [67]. Hydrogen bonding aids in a highly ordered structure. Hydrogen bonding between chains is very strong in crystalline regions, responsible for the high strength of fiber and making it insoluble in most of the solvents. In amorphous regions, these chains can bond with other molecules, such as water. Cellulose is hydrophilic, although it is insoluble in water; water absorption causes swelling. Cellulose properties are influenced by factors such as the type of plant, fiber modification, age of the plant, extraction methods, chemical composition, location of the plant, the maturity of the plant, and microscopic and molecular defects [66,68].

### 2.2. Hemicellulose

Hemicellulose is different from cellulose due to different sugar units. Hemicellulose is a branched non-crystalline or amorphous structure, different from a linear cellulosic structure. It has a degree of polymerization between 50 and 300, considerably less than cellulose. Hemicellulose acts as a compatibilizer to support microfibrils, cellulose, and lignin [69]. It is hydrophilic in nature, soluble in alkaline solution, and easy to hydrolyze in acids [64]. Hemicellulose may differ from plant to plant with different constituents [70]. Table 1 shows constituents of different natural fibers. 

## 3. Fiber Modification

Fiber modification helps to overcome various problems of natural fibers, such as poor fiber/matrix adhesion [35], moisture absorption [81], low fire resistance [82], inferior mechanical properties [83], low thermal resistance, and restrictive processing temperatures [32]. A wide number of methods are used to overcome these problems.

### 3.1. Fiber/Matrix Adhesion

Fiber addition in a matrix significantly alters the properties of the matrix due to the dependency of bio-composites properties on the fiber/matrix interface. Strong interface bonds must be ensured to achieve the majority of desired mechanical properties. Many physical properties are considerably improved with strong fiber/matrix adhesion [35]. A poor fiber/matrix interface results in reduced mechanical and physical properties. The hydrophilic nature of fiber is one reason for poor interfaces, which leads to poor fiber dispersion in a matrix. Hydrophobic matrix material and hydrophilic fibers are incompatible, which reduces the composite’s ability for stress transfer between the matrix and fiber. Fiber dimensional changes lead to microcracking, thereby affecting fiber/matrix adhesion [76,82,83]. Many techniques (e.g., surface treatments) have been employed to enhance fiber/matrix adhesion. In addition to improving fiber/matrix adhesion, surface treatments also reduce moisture sensitivity. Surface treatments employ methods such as solvent extraction, physio-chemical treatments, corona discharge, plasma discharge, laser, gamma-ray, and UV bombardment, and chemical modifications by the condensation of coupling agents on a surface or their placement by free radical technique [20,84]. Physical techniques modify the surface to increase interface bonding for better matrix and fiber adhesion [23]. Corona discharge modifies natural fiber’s surface energy and improves its compatibility with matrix material [85]. Tensile properties are significantly enhanced with the corona treatment of hemp fiber [86]. Plasma treatment also improves fiber/matrix compatibility. In plasma treatment, the charge is indued on the surface, and various gases can induce different modifications. Surface energy is improved due to surface cross-linking. Plasma-treated flax fibers have demonstrated enhanced fiber/matrix adhesion [87]. Interfacial adhesions were improved in jute fibers by oxygen plasma treatment with the induction of hydrophobic characters in fibers [88]. Chemical methods improve fiber/matrix adhesion by introducing new groups between incompatible fibers and matrices [23]. Silane treatment is an efficient method to improve fiber/matrix adhesion using SiH_4_ [89]. Maleated coupling improves a composite’s strength by improving the fiber/matrix interface [90]. Another method is a bacterial modification, which improves fiber/matrix adhesion through better mechanical interlocking. The use of cellulose produced from bacteria is considered a green method for surface modification and provides new means to modify fibers [91]. Alkali treatment or the Mercerization process is used to induce rough surfaces at the fiber/matrix interface [92]. The alkaline treatment works by altering hydrogen bonding in the structure and removing some lignin, waxes, and oils to expose short-length crystallites [93]. Alkaline-treated fiber has an increased concentration of exposed cellulose and surface roughness for better interlocking [94,95]. High alkali concentration has adverse effects on fibers, which can weaken and damage the fiber. Optimum concentration must be ensured to have most of desired mechanical and physical properties [96]. Benzoyl treatment is also used to improve fiber/matrix adhesion [97]. Detailed discussion on this method is present in the next section. Isocyanate with a functional group (−N=C=O) readily reacts with the hydroxyl group in lignin and cellulose to form strong covalent bonding. Isocyanate acts as a promoter and a coupling agent to provide better fiber/matrix adhesion [98]. Graft copolymerization is an effective surface treatment. Vinyl monomers are grafted on the surface, improving fiber/matrix adhesion [99,100]. Permanganate treatment forms reactive manganate ions. These ions react with hydroxyl groups in cellulose to initiate graft polymerization. This treatment provides chemical interlocking at the fiber/matrix interface to improve adhesion [101].

### 3.2. Reducing Moisture Absorption by Natural Fiber

Strong polarized hydroxyl groups make natural fibers more hydrophilic. They absorb most of the moisture from the surrounding environment [102]. The fiber cell wall has many hydrogen bonds. As the water comes in contact with the fiber, old hydrogen bonds break, and new hydrogen bonds are formed between hydroxyl groups and water molecules which are responsible for water absorption [97]. Hemicelluloses are mainly responsible for moisture absorption in natural fibers [103]. Hydrophilic natural fiber absorbs moisture, affects mechanical properties, gives dimensional instability, and develops internal stresses [32]. Capillary action and water intake fills voids in the composite, giving dimensional instability to the composite [20]. Moisture absorption causes swelling and microcracks [104]. The hydrophilic nature of fibers prevents the use of bio-composites in various potential applications [105]. Moisture absorption makes the composite a breeding ground for fungi, bacteria, and harmful insects [106]. Apart from water absorption, micro gaps and cracks can be a result of poor processing conditions, incompatibility between the fiber and matrix, and poor environmental and service conditions [107]. Water absorption in bio-composites is, somehow, a complex process due to the involvement of a hydrophobic matrix and hydrophilic fibers. Diffusion and percolation are two main mechanisms of water intake. In diffusion, water molecules are transported from higher concentration areas to low concentration areas due to their random motion [108,109], while in percolation, water is passed through pores of the composite [110]. Moisture absorption and composite swelling are directly proportional to fiber thickness and size [111]. Water absorption in composites decreases the Young’s modulus, the stress at maximum load, and various other mechanical properties [112]. Fiber must be modified physically and chemically to overcome moisture absorption issues. Compatibilizers and adhesion promotors showed promising results to reduce moisture absorption [113]. Hydrothermal treatment increases the crystallinity of cellulose in natural fiber and extracts hemicellulose content, effectively reducing moisture intake. The Duralin process is also employed to reduce the moisture content and swelling of the composite [114]. Acetylation of natural fiber is a renowned esterification method, originally used to prevent the cell walls of wood cellulose from water. Acetylation introduces the acetyl functional group (CH_3_COO^−^) with acetic acid as a byproduct [115]. In this method, acetic anhydride replaces hydroxyl groups responsible for the hydrophilic nature of fiber [116]. This method is useful to decrease the moisture absorption properties of the composite [117]. Acetyl-treated fibers exhibit better resistance to tensile strength loss during treatment [118]. Benzoyl treatment is another method used to improve fiber/matrix adhesion, thermal stability, and to decrease the hydrophilic nature of the fiber. Benzoyl chloride is used in fiber treatment. This method removes the majority of lignin and waxes, along with oily materials, to expose reactive hydroxyl groups at the surface. Benzoyl groups substitute hydroxyl groups, making natural fiber hydrophobic and improving adhesion properties [97]. Peroxide treatment is an effective method to reduce the moisture absorption in fiber. In this method, free peroxide radicals react with hydroxyl groups to achieve the desired results and to improve the thermal stability [119]. The sodium chlorite method has shown promising results to reduce moisture content and to enhance the hydrophobic properties of the fiber. Sodium chlorite is utilized in bleaching the fibers to form chlorine dioxide, which reacts with lignin to effectively remove it, and also reacts with hemicellulose to improve the hydrophobic nature [120,121]. Steric acid contains carboxyl groups, and these groups react with hydroxyl groups to decrease their hydrophilic nature. Pectin, waxes, and oily materials are also removed in this method [49,122]. Permanganate treatment releases manganate ions to react with hydrophilic hydroxyl groups of cellulose to improve water resistance natural fiber [123]. Triazine treatment uses triazine derivates with multifunctional groups to reduce moisture adsorption in fibers. Chlorine in multifunctional groups reacts with hydroxyl groups by esterification and provides a link between cellulose and the coupling agent to enhance hydrophobic properties of the fiber [124,125]. Fatty acid derivates are also used to improve the water-resistance of fibers. Oleoyl chloride is one of the fatty acids derivates which reacts with the hydroxyl group through esterification. Hydrophilic hydroxyl groups are removed by this method, giving hydrophobic characteristics to fibers [126]. Fungal treatment is considered one of the best methods to increase the water-resistance of a fiber. It is a green and eco-friendly method. Non-cellulosic components of fibers are removed by specific enzymatic actions, along with lignin and hemicellulose removal. Excessive enzymatic activity may lead to a decrease in fiber strength [127].

### 3.3. Thermal Degradation and Flammability Properties

Natural fiber constituents such as cellulose, hemicellulose, lignin, pectin, and waxes are responsible for degradation and poor thermal properties. Both thermal stability and moisture absorption properties are temperature-dependent [128]. Poor thermal properties lead to the degradation of fibers with the release of various volatile products [20]. Cellulose degrades between 260 °C and 350 °C, and hemicellulose between 200 °C and 260 °C, while lignin starts to decompose at 160 °C and continues to degrade up to 400 °C [18]. Natural fiber-based composites decompose with the release of toxic byproducts and heat [129]. Composite combustion releases toxic byproducts, smoke, char, combustible and non-combustible gases [130]. Properties such as thermal stability, fire properties and water resistance are dependent on the constituents of natural fibers. High cellulosic contents make natural fibers readily flammable [131]. Orientation and structural properties of fibers play a vital role in determining thermal and flammability properties. Flammability and thermal properties are improved with the addition of silica and ash [132].

High crystallinity, char production and low polymerization improve fire resistance. Char protects the materials’ core from combustion and maintains structural integrity [133]. Composites undergo heating, decomposition, flame ignition, combustion, and propagation during the burning cycle [134]. Due to poor flammability, natural fibers have been limited to a few applications [131]. It is a challenge to find methods to overcome this issue, because few studies have been reported to address this problem. Flax fiber has the best fire resistance properties due to low lignin contents [135]. Thermal stability and the flammability of natural fibers is studied through various techniques, such as thermogravimetric analysis (TGA), vertical flame tests, cone calorimetry techniques, etc. The rate of flame spread, heat rate reserve (HRR), mass loss, and carbonization rate impact the flammability of fibers [82]. Some of the methods to minimize flammability and thermal issues are: the use of nanoparticles, fire retardant coatings, impregnation of natural fibers with fire retardants before manufacturing, the use of non-flammable binders, resins, polymer matrices, and the insulation of composites to prevent possible damage from heat or flame [136,137]. Ammonium, halogens, boron, phosphorous, bromine, aluminum and magnesium-based compounds, zinc borate, silica, graphite, and alkaline earth metal compounds are commonly used fire retardants. Phosphorous-based fire retardants exhibit auto-extinguish behavior in a composite [2,134,138]. Ammonium-based retardants produce char, while bromine-based compounds terminate chemical reactions for combustions [130]. Due to toxic byproducts, the use of halogens is not recommended [138]. Phosphorous-based fire retardants are not preferred due to environmental hazards and health issues [139]. Nanoparticles and nanocomposites have shown good flame and thermal resistance properties but are not cost-viable. The addition of flame retardants improves flammability properties by increasing the specific heat and thermal conductivity, preserving physical integrity, and reducing combustion heat in bio-composite [130]. The addition of flame retardants may have some adverse effects, such as poor fiber/matrix adhesion and the poor dispersion of fibers in a matrix [139]. Table 2 contains various research studies for bio-composites and surface modifications.

### 3.4. Mechanical Properties

Bio-composites are used in the automobile, construction, and packaging industries. Bio-composites are mostly used in non-load-bearing and non-structural applications due to limitations in mechanical properties [150]. The construction sector requires composites to bear high stress, compression, and tension [129]. Natural bio-composites exhibit reasonable mechanical properties such as stiffness, strength, flexibility, and Young’s modulus [151]. Fiber type, fiber orientation, microfibril angle, treatment type, physical properties, and adhesion between the fiber and the matrix are essential characteristics in composites to determine mechanical properties [152]. The microfibril angle determines the stiffness of the fiber [18]. Natural fibers act as reinforcements to improve mechanical properties [152]. Fiber/matrix adhesion is the most critical factor for the determination of mechanical properties. Better adhesion improves the stress/load transfer between fiber and matrix. Tensile strength is mostly dependent on matrix properties, while modulus is dependent on fiber properties [153]. Mechanical properties of different fibers are listed in Table 3.

## 4. Biodegradable Matrix Materials

Biodegradable matrices are environmental and eco-friendly. Disposal and the environmental problems of composites can be solved using renewable and biodegradable matrices and fibers [168]. These biodegradable composites have given rise to new markets [169]. Biodegradable polymer matrices are categorized according to the source. Biodegradable polymers are created through agricultural products, byproducts, microbial actions, and chemical methods [26,170]. Bio-composites are fabricated by combining natural fibers in a matrix material. The matrix material can be biodegradable, non-biodegradable, or synthetic. Synthetic matrix materials, along with natural fibers, are used to form hybrid bio-composites [18]. Polymers can also be fabricated by combining or blending two biopolymers. The biodegradability of the polymer depends on factors such as the origin of the polymer, the polymer structure, and the conditions around the polymer during degradation [171]. During biodegradation, the biopolymer is decomposed by microbial actions with the release of CO_2_, various compounds, and biomass [161]. Biodegradable polymer matrix materials have poor properties, but the introduction of natural fibers increases mechanical properties [172]. Conventional polymers such as polyethylene, polyester, polypropylene, and epoxy have been through the cycle of development and commercialization [173,174,175]. Years of research and development have resulted in high performance and better mechanical properties. However, recycling and environmental issues have shifted attention to bio-polymers [176]. As of 2020, a total of more than nine billion metric tons of synthetic polymers has been produced from petroleum products [177,178]. Only about 25% of total produced synthetic polymers are recycled or incinerated; most of it ends up in landfill sites or the natural environment [179]. Low mechanical properties, lack of manufacturing processes, high production cost, low melting temperatures, and a narrow processing temperature range are limitations of biopolymers. Several methods have been employed to produce higher molecular weight polymers [180,181]. Bio-based polymers can replace synthetic polymers in key sectors such as the construction, automobile, and packaging industries.

Some of the examples of bio-based polymer matrices are polybutylene succinate (PBS), polylactic acid (PLA), poly hydroxyalkanotes (PHA), and poly(*ε*-caprolactone) (PCL) [9,37,38,39].

### 4.1. Polybutylene Succinate (PBS)

Polybutylene succinate (PBS) is fabricated from succinic acid and 1,4-butanediol (BDO). PBS is a biodegradable polymer with excellent mechanical and thermal properties [182]. PBS belongs to the aliphatic polyester family. Due to ever-increasing environmental concerns, monomers from renewable sources are encouraged for use [183]. Succinic acid is produced from maleic anhydride hydrogenization: maleic anhydride is converted into succinic anhydride; and highly pure succinic acid is produced via the electrolysis route. Highly pure succinic acid is required in the pharmaceutical and food industries [184]. Succinic acid is also produced through the fermentation of agricultural carbohydrates and byproducts [185]. Microbial organisms are responsible for fermentation. Different bacterial cultures are used in fermentation processes, such as *Actinobacillus succinogenes* and *Anaerobiospirillum succiniciproducens* [186]. The U.S.A. Department of Energy recognized succinic acid as a replacement for petroleum-based products. Succinic acid is used in the preparation of different chemical compounds [187]. BDO is produced mainly from petroleum products [184]. Various processes such as the Reppe process, Mitsubishi 1,3-butadiene acetoxylation technology, the LyondellBasell propylene oxide route, and Davy Process Technology are used in industries [183,188]. PBS is synthesized through two-step polycondensation. The first step involves esterification and transesterification of succinic acid and BDO [189]. Oligomers are produced by esterification, and the next step involves removing BDO via polycondensation to form higher molecular weight PBS. In this process, the reactor must have a mechanical stirrer, oil bath, and nitrogen gas [190], along with various catalysts [190].

PBS is a highly crystalline polyester with a melting temperature of around 115 °C, critical for high-temperature applications [191]. Tensile yield strength and Young’s modulus reach up to 35 MPa and 500 MPa, respectively. Both properties are highly dependent on the degree of crystallinity for PBS [183,192,193]. Physical properties of PBS are altered by copolymerization with different comonomers, such as ethylene glycol and adipic acid [192,193,194]. Aliphatic structure and the presence of ester bonds makes PBS biodegradable in lipase mixtures, soil, moisture content, and sludge [41,182]. Biodegradation of PBS depends on chemical structure, the degree of crystallinity, specimen size, and conditions around the specimen. The rate of aliphatic polyester is slowed down with an increase in aromatic content, while it increases with the addition of comonomers [193,194,195]. The degradation rate is controlled by PBS copolymerization. Processability of PBS is strongly dependent on melt strength, the viscosity of the melt, thermal stability, the degree of crystallization, and water resistance. Extrusion and injection molding are suitable for PBS fabrication for molecular weight under 100,000, while blowing and casting mechanisms require higher molecular weight PBS for easy processing [183]. Mechanical properties of PBS are improved significantly by blending with other bio-based polymers such as starch, PLA, carbohydrates, and its copolymers [194]. Tensile, elastic modulus and impact properties of PBS are enhanced with polylactic acid (PLA). With some limitations at the molecular level, PBS and PLA are compatible [196]. PBS is replacing conventional polymers in packaging, bottles, molds, fibers, flushable hygiene, shopping bags [41,197], etc.

### 4.2. Polylactic Acid (PLA)

Polylactic acid is an eco-friendly, bio-degradable polymer and is made up of lactic acid produced from renewable agricultural products and byproducts such as starch extracted from potatoes, corn grain, sugar cane, etc. Hydrolytic or thermal degradation of PLA results in low toxicity byproducts [198,199,200,201]. Lactic acid is categorized into two optically active isomers, l and d type enantiomers [202,203]: l type lactic acid rotates the polarized light in a clockwise plane; d types rotate anticlockwise. Isomer type dictates PLA classification in a family of three polymers: poly-d-lactic acid (PDLA), poly-l-lactic acid (PLLA), and poly-l-d-lactic acid (PDLLA) [201,204]. PLA is fabricated through three routes, which are polymerization through the formation of lactide, direct condensation polymerization, and azeotropic dehydration condensation [205,206,207]. Ring-opening polymerization (ROP) is one of the most widely used methods to fabricate PLA [208,209]. A cyclic dimer of lactic acid is used in ROP, and the process involves ring-opening of the cyclic dimer in lactic acid.

Catalysts are used to control the molecular weight of PLA [210]. The l:d ratio in lactic acid is controlled through factors such as temperature, residence time, type, and concentration of catalyst [203]. ROP is performed in bulk, melt, or even in solution form through different mechanisms such as cationic, anionic, and coordination insertion [211]. The direct or polycondensation route is the least expensive PLA process—it involves solution and melts polycondensation with different solvents under high vacuum and temperature [200,202]. Limitations in the production of solvent-free high molecular weight PLA are associated with these routes [212]. Condensation polymerization yields low molecular weight PLA, making it unfit for many applications. A low molecular weight in PLA is due to polymer melt viscosity, low concentration of reactive end groups, moisture presence, and impurities. Coupling agents or esterification promotors increase molecular weight but with the addition of increased complexity and cost. Mechanical properties and molecular weight are improved by removing solvents, moisture, and impurities from the melt. Azeotropic dehydration condensation is used to obtain high molecular weight PLA without any use of coupling agents or esterification promotors. Water is removed to produce high molecular weight PLA [200,209,212]. This process involves the distillation of lactic acid for 2 to 3 h at a temperature around 130–140 °C with the use of a catalyst. Catalysts can cause impurities and an increase in production costs. Catalyst impurities are removed with the addition of various acids. Enzymatic polymerization is another method to produce PLA with mild processing conditions [200,208,213].

Research studies are trying to reduce the shortcoming and limitations of PLA [213]. PLA requires an estimated 25–50% less energy in fabrication than conventional polymers, contributing to cost reduction in the process [214]. PLA has high strength, processability, and mechanical properties. PLA exhibits better tensile properties than polystyrene PS and polyethylene terephthalate (PET) [215]. PLA is the better choice for tensile and flexural modulus properties among PET, PP, and HDPE. Impact strength values and elongation at break are lower than PET, PP, and HDPE [216]. PLA is crystallized in three structural forms: α, β, and γ. α form is developed from a melt or cold crystallization, and mechanical stretching and deformation in the formation of the β form. PLA has a high crystallization rate due to fast-growing semi-crystalline regions [217,218]. Crystallinity influences properties such as tensile strength, melting temperature, hardness, stiffness, etc. PLA is brittle with low toughness. Low toughness values limit the use of PLA in high-stress level applications. The inertness of PLA causes troubles in surface modifications for various applications [201,207]. Thermally unstable PLA loses molecular weight during thermal treatments and processing temperatures. Degradation of ester linkages, even at a temperature lower than melting temperature, causes this thermal instability. Degradation increases by many folds after melting temperature. Several methods are used to overcome this issue, such as hydrolysis, oxidative degradation, esterification reactions, and depolymerization. Thermal degradation is dependent on factors such as moisture content, size of the particles, lactic acid concentration, the molecular weight of PLA, impurities, and the catalyst used [207,219,220]. PLA is degradable by hydrolysis when PLA is exposed to moisture for longer periods. Degradation has two main steps. The first step involves the degradation of ester groups and the lowering of PLA molecular weight. The second step involves yielding CO_2_ and water through microbial action to degrade low molecular weight oligomers [212,219]. PLA is blended with other polymers to overcome limitations and improve mechanical properties, degradation, and thermal instability [203]. PLA is used in various household applications, such as plastic bags, sanitary products, cups, bottles, plates, etc. [198]. PLA applications range from textile products, to the pharmaceutical, medical, and packaging industries. In biomedical applications, PLA eliminates the need to remove medical implants due to biological degradation with time. PLA has good compatibility with human tissues and organs and has low production costs and excellent mechanical properties. PLA is used for surgical sutures in tissues, skin, and closing wounds. PLA is additionally used in drug delivery systems for the continuous release of drugs [207,208,220,221]. The non-toxic degradability of PLA is very effective for implants and supports in the human body. PLA may take four months to four years to degrade completely, dependent on chemical composition, crystallinity, polymer porosity, and the degradation environment around the implant. PLA is used for bone fixation devices, replacing metallic fixation devices. PLA is readily used to make screws, pins, and wires for bone fixation [220,221]. PLA provides polymeric support in tissue engineering and is used in tissue or organ construction. PLA is used to develop filaments to reconstruct nerves for paralyzed persons. Schwann cells are grown on PLA polymeric supports. Schwann cells are grown in damaged nerves to join them artificially. Over time, polymeric support degrades, forming nerve connections. Adhesion of Schwann cells on PLA support is limited and can be increased by various plasma techniques [222,223,224]. Surface properties of polymers play vital roles in biomedical applications. Various techniques have been utilized to improve surface properties. These techniques include different physical and chemical methods, along with plasma and radiation treatments. PLA is also used to produce agricultural equipment and disposable materials, in the automobile industry, and textile industry [198,199,200].

### 4.3. Poly Hydroxyalkanote (PHA)

PHA belongs to a group of *R*-hydroxyalkanoic acids. PHA is an eco-friendly and renewable polymer [225]. Only a few among 150 hydroxy acids monomers actively produce PHA under normal conditions. The short-chain PHA has three to five carbon atoms, which improve stiffness, brittleness, and crystallinity. Middle chain polymers have six to fourteen carbon atoms and have a low degree of crystallinity [226,227]. PHA is dependent on plants and bacteria for mass production [228]. Carbon accumulation in cells influences PHA microstructure and properties [229]. PHA is also produced using Gram-positive and Gram-negative bacteria, with over 75 different types, such as *Pseudomonas*, *Bacillus*, and *Ralstonia* [227]. Bacterial PHA has a high molecular weight, varying between 200,000 to 30,00,000 Da due to different bacteria types and growth conditions [230]. Carbon accumulation in a cell is the source of PHA [231], which only happens under stressed conditions with a limited supply of nitrogen, oxygen, and phosphorus nutrients. Large-scale microbial fermentation is used for the mass production of PHA. Mass production is dependent on factors such as bacteria type, cell density, growth rate of bacterial strain, total process time, substrate, and purification methods [231]. Sugar, fatty acids, sucrose, starch, molasses, wheat, corn, methane, and activated sludge effluent substrates are used in PHA production [232,233,234,235]. PHA is commercially produced through glucose and sucrose substrates [236]. High production costs, processing constraints, and poor mechanical and physical properties are some of the factors limiting PHA applications [237]. High output is achieved through batch or continuous fermentation processes. PHA is produced in two-step continuous fermentation. In the first step, biomass is provided with the full amount of nutrients, while limited nutrients are provided in the second step. Some bacteria are efficient in limited nutrient supply, and vice versa. A well-balanced cell growth rate of bacteria is essential to avoid the premature ending of the fermentation process [230,238,239,240,241]. PHA is stored in cells that have to be extracted; however, extraction methods have cost limitations [242]. Solvent extraction is the simplest method for PHA extraction, where a cell is ruptured to access PHA. PHA dissolves in selective solvents and is precipitated by non-solvents. Chlorine solvents are used to dissolve PHA, while ethanol and methanol are used for precipitation [243]. A floating method in solvents is employed to recover highly pure PHA [244]. The digestion method is an alternate solvent extraction method that involves chemical or enzymatic digestion mechanisms. Chemical methods degrade polymers and have harmful byproducts, thus, reducing the overall extraction efficiency [245]. Enzymatic digestion methods are target-specific and are highly efficient in PHA extraction [243]. Modern technology supercritical fluid extraction methods are environmentally safe and cost-effective. Diffusion rates are enhanced due to the low viscosity of supercritical fluids having negligible surface tension [246]. CO_2_, methanol, and ammonia are used as supercritical fluids [247]. Parameters such as temperature, pressure, and impurities determine the supercritical fluids’ efficiency. The aqueous two-phase method consists of two different and unique phases and extracts high purity PHA [248].

PHA is a degradable polymer, and the degradation rate is dependent on the surrounding environment, pH level, water content, chemical composition, crystallinity, and surface area of PHA [230]. Micro-organisms degrade biopolymers with the release of hydroxy acids, which are carbon sources. PHA degradation releases CO_2_, water, and methane. PHA is degradable up to 60 °C [229] and used in bone and tissue engineering [226,249]. PHA is used in the development of heart valves to replace metallic valves [250], and also in drug delivery systems; PHA acts as a carrier for drugs to transport bio-active compounds to the target area [251]. PHA shows promising results in nerve grafting to repair damaged nerves [252]. Thus, with enhanced compatibility and adhesion with natural fibers, PHA is used as a polymer matrix in bio-composites [253].

## 5. Processing Techniques of Bio-Composites

Bio-composites are gaining momentum in various industries and research studies. The demand for bio-composites has been tremendously increased in domestic and industrial sectors due to a surge in environmental pollution concerns and increased environmental regulations by local and international bodies. For desired properties to achieve and make bio-composites cost-effective, different modified chemical treatments and efficient process techniques are needed [64]. Bio-composites are currently fabricated through conventional methods such as compression molding, hand lay-up, injection, extrusion, and pultrusion [254]. These manufacturing techniques are the result of years of research and development. Research studies focus on developing and modifying existing techniques to increase the quality of bio-composites and make them cost-effective. With a few modifications in existing techniques, bio-composites can be readily fabricated [23]. Fabrication techniques are selected based on the requirements for fiber dispersion, orientation, and aspect ratio in desired applications [255]. Factors such as production cost, final design, shape, size, raw material properties, and process constraints are taken into account in selecting any process and technique. Fiber/matrix adhesion, uniform dispersion of fibers, and a high aspect ratio enhance the mechanical properties of bio-composites. Some of the factors affecting manufacturing techniques and properties are fiber length, content, type, orientation, and moisture content. Proper drying of fibers is necessary because moisture can alter properties and process parameters. Moisture in fibers increases void content and porosity in bio-composites, which affects the final mechanical properties [256,257].

Various modification methods are employed to overcome the moisture issue in fibers [23]. The presence of compounds such as silica and the chemical structure differences among fibers affects manufacturing processes and properties in bio-composites [258,259]. Natural fibers degrade at a high temperature, which gives a narrow temperature range in composite manufacturing [260]. Bio-composites must have a good shelf life and structural integrity while in service [26]. Due to their non-abrasive nature, natural fibers cause less wear and damage to tools and machines used in manufacturing [261]. Induced stresses solidify melt prematurely, and end products can be shrunk by up to 8% [262]. Excess fibers bundle up to form agglomerates and adversely affect bio-composites’ final properties [260]. Premature solidification and loss in the strength of final products are also caused by high viscosity of the melt, while homogeneity of final products is affected by fiber length [263].

The addition of various additives solves processing issues, but the overall cost increases and can cause new issues.

### 5.1. Compression Molding

Compression molding is a reliable method due to the high production rate and low processing time. Compression and flow compression molding is employed in this process. These two techniques are differentiated based on the final products. Glass fibers are manufactured through compression molding, and similar molding techniques can be applied in natural fiber composites. Bio-composites fabricated through this process have adequate mechanical properties for different applications [264]. Compression molding is used for bulk production, such as in automobile parts production [265]. Compression molding decreases fiber strength due to the dependency on initial fiber length and various process parameters such as melt viscosity and screw speed and design. The incompatibility of natural fibers with matrices also reduces fiber strength and the strength of bio-composite [266]. In compression molding, fibers are placed between matrix layers.

Furthermore, load and heat are applied in the process [267]. Compression molding is categorized into hot pressing and auto-clave methods. Sheet and bulk molding materials are starting materials used to cover around 30–70% mold cavity. The mold is closed correctly along with the application of pressure and heat. Natural fibers may break due to high pressure and temperature. Sometimes, short fibers are mixed prior to compression molding to reduce shrinkage and increase the strength of final products [268].

### 5.2. Extrusion

The plastic industry mostly uses extrusion. Single screw extruders or double screw extruders are used, and screws are rotated clockwise or counterclockwise depending upon the final products. For the limited mixing of melt, a single screw is used. In contrast, a twin-screw extruder is used for the vigorous mixing of melt. Natural fibers are dispersed uniformly in the melt by a twin-screw extruder. Extrusion is a hot-melt technique used for the continuous production of bio-composites [62]. Single and twin extruder have a different processing temperature range, process parameters, and screw designs. Process parameters influence mechanical and thermal properties [269]. Process parameters such as high pressure and high temperature damage natural fibers, albeit resulting in a better aspect ratio but at the cost of mechanical properties such as high porosity [270]. Hang et al. [269] studied the mechanical properties of composites made up of flax fiber using a twin-screw extruder. Chaitanya et al. [271] studied the processing of PLA/sisal bio-composites using extrusion injection molding. The final composite had a 30% weight fraction of fibers. Extrusion injection molding is suitable for processing short and long length fiber, but breakages were observed for long fibers. Extrusion exhibited uniform fiber dispersion for homogenous bio-composites with better mechanical properties. Ranganathan et al. [272] studied the structural properties of jute and viscose fiber hybrid composites. The effect of fiber length and fiber content on fracture toughness and fatigue properties were studied. Polypropylene was used as the matrix material in twin-screw extrusion. Jute fibers were dried at 60 °C for 48 h, and viscose fibers were dried at the same temperature for about two hours. Samples of 30 wt.% jute fiber with the polypropylene matrix and various weight percentages of viscose fibers were prepared. The composite with 30 wt.% jute fiber showed unstable crack growth with lower fracture toughness. The addition of 10 wt.% of viscose fiber stabilized crack growth. The fatigue life of the composite with 10 wt.% of viscose was three times higher than that of jute fiber composite. The addition of viscose fiber increased fracture energy. Awal et al. [273] analyzed PLA and cellulose fiber composites for thermal and mechanical properties. Bio-composite was fabricated using extrusion molding. Wood cellulosic fibers were used after drying fibers at 80 °C for 24 h. Thermo-gravimetric analysis exhibited an upper limit for processing temperature. The heat distortion temperature was slightly improved for the high-temperature life cycle for PLA and wood fibers composite. The addition of 1.3 wt.% BioAdimide significantly improved the tensile strength and impact strength of the composite. Bio-additives improved the interactions between the fiber and the matrix for better mechanical properties and processability of bio-composites. Tensile modulus for the PLA/wood/adimide composite improved by 26% from the PLA/wood fiber composite.

## 6. Applications of Bio-Composites

A small number of bio-composites are commercialized and developed. Most of the bio-composites are still under research and development. New processing techniques and technologies are being developed to produce bio-composites at a lower cost. Mostly, bio-composites are used in non-structural and non-load-bearing applications. Developing countries are abundant in natural fibers, but the lack of resources prevents using these fibers in composites and developing new processing techniques, while developed countries in Europe and Asia are ahead in the development of bio-composites [274]. Despite the benefits of these bio-composites, some challenges such as cost reduction, reliable performance, and inferior mechanical properties are still to be addressed for mass production [26]. Despite these challenges, bio-composites still have great potential to be used in various applications. Research has shown promising results, but more research and developments are required to commercialize bio-composites successfully [67]. Focus is being paid to achieve properties comparable with synthetic composites. Bio-composites are biodegradable, renewable, and natural composites with minimum impact on the environment and considerably lower carbon emissions [275]. Growing awareness among people and new laws for environmental protection will promote meaningful improvements for bio-composites. Additionally, developments in agricultural sciences will help to harvest fibers with more favorable properties for these bio-composites. In the near future, bio-composites may completely eradicate the dependence on synthetic products [276]. The energy required for the production of bio-composite is much less than that of synthetic fiber. The production of synthetic composites is energy extensive, while bio-composites save energy [7]. Different governments are encouraging industries to use bio-degradable materials to overcome waste and pollution-related issues [277]. One of the main drawbacks in the use of bio-composites is the variation of mechanical properties in plant fibers. Change in the region, climate, and even fiber from another planet of the same type will likely be different in properties [278]. These shortcomings are balanced through different processing and chemical treatments. The automobile, construction, textile, and packaging industries are the primary industries to employ bio-composites.

### 6.1. Automobile Industry

Conventional composites have glass and carbon fiber reinforcements that have so far dominated the automobile industry. Renewable alternatives are required to address environmental concerns and reduce petroleum-based composites’ carbon footprints [279]. Several candidates have been studied, exhibiting promising results, while others are in the development phase. Various natural fibers such as flax, hemp, kenaf, jute, coir, and sisal are used to produce bio-composites for automobiles. Bio-composites are also used in automobiles to reduce overall weight, cut down production costs, and improve fuel efficiency. Bio-composites are used to produce different components, such as bumpers, door panels, seat pads, cup holders, trunk covers, armrests, headrests, and seat pads. Furthermore, bio-composites are known to reduce vibrations and noise through damping [264]. Ford uses soy foam seats, bio-based cushions, and hemp fiber composites in the front grills in various vehicle models [280]. Similarly, Mercedes-Benz use jute-based bio-composites for interior panels, flax fiber composites for shelves and trunk covers, and sisal-based composites for rear panel shelves [281]. The use of bio-composites led to a reduced weight of roughly up to 10%, and energy consumption up to 80%, compared to synthetic composites. Toyota use kenaf fibers in tire covers, soy foams for vehicle seats, and PP/PLA-based bio-composites inside trims, toolbox areas, and package trays [282]. Similarly, Volkswagen use bio-composites to make door panels, flap linings, door inserts, and package trays.

### 6.2. Construction and Textile Industry

In the construction industry, bio-composites are used to manufacture windows, doors, window frames, ceilings, floor mating, and roof tiles. Load-bearing applications include the manufacturing of floor slabs, beams, pipes, and tanks. Furthermore, bio-composites are employed in the repairing and rehabilitation of various structural components. Due to better thermal and acoustic properties, natural fiber composites are used as insulating and soundproofing materials [283]. Hemp/lime/concrete composites have exhibited better sound absorption ability than any other binders [284]. Life cycle assessment, durability properties, and ecological aspects are taken into account before selecting any bio-composite as a construction material. Low weight and comparable mechanical properties with synthetic composites are crucial for construction applications. Similarly, natural fiber composites have enormous potential to be used in the textile industry to manufacture ropes, sacks, bags, and clothes. Moreover, many countries are adopting bio-composite materials to address environmental issues. Many industries are investing in bio-composites due to future demand.

## 7. Conclusions

The potential of bio-composites to be used as eco-friendly, renewable, and sustainable substitutes is the main driving force for research, development, and commercialization. The use of bio-composites in various applications has opened avenues for research studies and industries to explore further. Early on, the lack of fabrication methods and higher production costs restricted bio-composites’ growth, but environmental issues have removed these hurdles. Bio-composites are regarded as the best replacement for synthetic composites because of their comparable mechanical properties and eco-friendly nature. Synthetic composites cause pollution, emit toxic byproducts, use excessive energy, and have recyclability issues and high carbon footprints. The sustainability of synthetic composites comes into question due to the depletion of finite petroleum resources. The use of synthetic composites must be limited to protect the environment. In this review, bio-composites were analyzed to provide an overview of the contemporary developments.

The structure, morphology, content, and mechanical properties of natural fibers were discussed in detail, along with natural fiber constituents. Micro-fibrils, lumen, and different bonding structures play important roles in determining the mechanical properties and low density of fibers. Different modification techniques to improve shortcomings such as the fiber/matrix adhesion, hydrophilicity, and flammability of natural fibers were employed. Modification techniques enhance fiber/matrix interlocking, as well as moisture and thermal resistance. Some of the degradable polymer matrices are polybutylene succinate (PBS), polylactic acid (PLA), poly hydroxyalkanotes (PHA), and poly(*ε*-caprolactone) (PCL). During biodegradation, biopolymers are decomposed through microbial actions with the release of CO_2_, various compounds, and biomass. The addition of natural fibers to these bio-degradable matrix materials enhances strength and other properties. Bio-composites are manufactured through conventional methods such as compression molding, hand lay-up, injection, extrusion, and pultrusion. Some of these manufacturing techniques and research studies are focusing on the development and modifications of existing techniques to increase the quality of bio-composites. Bio-composites were analyzed in terms of production cost, final design, shape and size, raw material properties, and process constraints. Various applications of bio-composites include construction, automobile, and textile industries. With the ever-increasing demand for bio-composites, numerous new potential applications for bio-composites will be developed in the near future.

## Figures and Tables

**Table 1:** Constituents of different natural fibers.

Fiber	Cellulose (%)	Hemicellulose (%)	Lignin (%)	Pectin (%)	Wax (%)	Reference
Abaca	56–63	21–25	7–12	0.8	3	[23,71,72,73]
Alfa	45.4	38.5	14.9	-	2	[71,72]
Areca	57.35–58.21	13–15.42	23–24	-	0.12	[74]
Bagasse	32–44	19–24	22	10	-	[71,75]
Bamboo	26–43	20.5	21–31	-	-	[23,71]
Banana	62–64	12.5	5–10	4	-	[31,75,76,77]
Barley	31–45	27–38	14–19	-	2–7	[31]
Coir	45.6	20	45	4	-	[71,72,78]
Corn	38–40	28	7–21	-	3.6–7	[31]
Cotton	82.7–90	4	0.75	6	0.6	[71,72]
Curaua	70.7–73.6	9.9	7.5–11.1	-	-	[23,71,72,75]
Eucalyptus	41.7	32.56	25.4	8.2	0.22	[79]
Flax	62–72.5	14.5–20.6	2.5	0.9	-	[23,71,75]
Hemp	81	14–22	4–13	0.9	0.8	[23,71,80]
Henequen	60–77.6	28	8–13.1	-	0.5	[71,72]
Hibiscus	28	25	22.7	-	-	[81]
Isora	74	-	23	-	1.1	[71]
Jute	59–71.5	12–20	9–13	0.2	0.5	[23,71,82,83]
Kenaf	53.5	21–33	17–21.5	2	-	[71,78,84]
Phromium	67	30	11	-	-	[71]
Pineapple	80.5	17.5	8.3–12.7	4	-	[23,71]
Ramie	72	5–16.7	0.6–0.8	2	-	[71,80]
Rice husk	28–36	23–28	12–14	-	14–20	[81]
Sisal	60–73	11.5–14	8–11	1.2	-	[23,71,85]
Sorghum	27	25	11	-	-	[31]
Wheat	33–38	26–32	17–19	-	6.8	[79]

**Table 2:** Different research studies for bio-composites and modification techniques.

Composite	Fabrication Method	Key Findings and Mechanical Properties	Effect of Surface Treatments	References
Abaca–Roselle/Cardanol formaldehyde composite	Compression molding	Natural fibers improved thermal, wear resistance, and mechanical properties of the composite and improved the hardness, density, and tensile strength of the matrix material. Tensile and flexural properties improved due to the presence of carbon and silica.	Alkali treatment increased fiber/matrix adhesion due to the removal of impurities and increased mechanical properties.	[140]
Areca fibers/Pine resin composite	Solvent casting method	The tensile strength of the composite is affected by the adhesion of the fiber/matrix; 10 wt.% areca fibers and 90 wt.% pine resin exhibited better mechanical properties due to efficient stress transfer between fibers and matrix.	Alkali treatment increased fiber/matrix adhesion. Tensile strength increased by 25%, while impact strength increased up to 24% due to treatment.	[141]
Banana fibers/PLA/Nanoclay composite	Melt blending	Nanoclay and PLA improved composite stability, flame resistance, and thermal properties. Nanoclay formed a protective layer at the surface to prevent flame and acted as a thermal barrier to prevent degradation.	Silane treatment improved fiber/matrix adhesion by increasing the contact area of fibers.	[142]
Flax/epoxy composite	Vacuum infusion	Flax/epoxy composite is suspectable to water absorption due to high void content.	Sodium bicarbonate-treated fibers had less void content mainly due to the removal of impurities. With the increase in sodium bicarbonate concentration in fiber treatment, properties such as flexural, tensile strength and flexural moduli increased.	[143]
Hemp fibers/polycaprolactone bio-composite	Twin screw extrusion	Flexural, tensile and impact properties of composite are improved. With the increase in aspect ratio of hemp fiber, water absorption increased. Flexural strength increased by 169% and flexural modulus increased by 285% for the aspect ratio of 26. Hemp fibers increased the stiffness of the composite.		[144]
Jute fibers/unsaturated polyester resin	Hand lay-up and compression molding	Jute fibers enhanced properties such as tensile, flexural strength, flexural modulus, and interlaminar shear strength. Untreated fibers lead to low density and low volume fraction.	Alkali-treated fibers showed an increase in tensile, flexural strength, flexural modulus, and interlaminar shear strength due to better fiber/matrix adhesion. Alkali treatment removes hemicellulose and increases interlocking points in fibers for better adhesion and stress transfer.	[145]
Jute fibers/clay/epoxy bio-composite	Compression molding	The addition of 15 wt.% clay improved mechanical properties due to uniform dispersion in a composite. Clay can agglomerate, which increases composite porosity and decreases fiber/matrix adhesion.	Alkali treatment improved fiber/matrix adhesion with increased cellulose after removing pectin, lignin, and other impurities. An increase in cellulose content leads to better interfacial adhesion.	[146]
Kenaf fibers/sea urchin spike filler/neem oil/epoxy composite	Hand lay-up	Neem oil made epoxy eco-friendly while sea urchin spike filler and kenaf fibers increased the toughness of the composite. The addition of neem oil leads to the formation of an interpolymer-penetrating network and ketone groups, which decreased hardness and overall tensile strength of the composite.	Amino silane-treated particles dispersed well in matrix material without agglomeration, which improved wear resistance and thermal degradation. Treated fiber formed a layer at the fiber/matrix interface, and high temperature was required to break this layer. Modified fibers increased the moisture resistance in the composite.	[147]
Ramie fibers/PLA composite	Hot compression molding	Low temperature and pressure in compression molding had led to poor fiber/matrix adhesion and wettability.	Alkali/silane-treated fibers composite had better tensile strength, modulus, and impact strength. Cellulose content increased due to the removal of impurities from fibers, which improved mechanical properties. Treated fibers had better stress transfer due to the formation of covalent bonds between fibers and matrix.	[148]
Sisal fibers/starch composite	Hot pressing	Compressive and tensile strength of the composite increased with the addition of sisal fibers. The addition of natural fibers increased the biodegradability properties of the composite.	Alkaline treatment increased fiber/matrix adhesion, which improved mechanical properties.	[149]

**Table 3:** Mechanical properties of different natural fibers.

Fiber	Density (g/cm^3^)	Diameter (µm)	Micro-Fibrillar Angle (°)	Moisture Content (%)	Tensile Strength (MPa)	Elongation at Break (%)	References
Abaca	1.5	10–30	20–25	5–10	400–980	3–10	[23,75,154,155]
Areca	0.7–0.8	-	-	-	147–322	10.2–13.15	[156,157]
Bagasse	1.25	10–34	-	-	222–290	1.1	[23,75,158]
Bamboo	0.6–1.11	240–330	-	9.16	140–800	1.40	[23,72,159,160,161]
Banana	1.35	50–250	11–12	10.71	529–914	3	[80,161,162,163]
Coir	1.2–1.5	100–450	30–49	8–11.36	175–180	30	[18,40,74,75,159,164]
Cotton	1.5–1.6	12–35	-	7.85–8.5	287–597	7–8	[72,163,164,165,166,167]
Curaua	1.4	170	-	-	500–1150	3.7–4.3	[23,161]
Flax	1.5	5–38	5–10	1.2–8	345–1035	2.7–3.2	[23,161,167,168,169]
Hemp	1.48	-	2–6.2	6.2–12	690	1.6	[23,161,163,164,169]
Henequen	1.2	-	-	-	430–570	3.7–5.9	[71,72]
Isora	1.2–1.3	-	-	-	500–600	5–6	[71,170]
Jute	1.3–1.5	20–200	8	12.5–13.7	200–773	1.5–1.8	[23,75,164,168,171]
Kenaf	1.4	70–250	2–6.2	6.2–12	930	1.5	[23,165,163,165]
Nettle	1.51	20–80	-	11–17	650	1.7	[75,155,167]
Oil Palm	0.7–1.55	150–500	42–46	-	80–248	3.2	[23,75,165,172]
Palf	0.8–1.6	20–80	14	11.8	180–1627	1.6–14.5	[74,75,112]
Piassava	1.4	-	-	-	134–143	7.8–21.9	[74,75,173]
Pineapple	0.8–1.6	8–41	-	10–13	170–1627	2.4	[23,162,167,174]
Ramie	1.5	50	69–83		220–938	2–3.8	[23,163,164]
Sisal	1.5	50–300	-	11	511–635	3–7	[23,163,164]
